# Drought Impact Is Alleviated in Sugar Beets (*Beta vulgaris L*.) by Foliar Application of Fullerenol Nanoparticles

**DOI:** 10.1371/journal.pone.0166248

**Published:** 2016-11-10

**Authors:** Milan Borišev, Ivana Borišev, Milan Župunski, Danijela Arsenov, Slobodanka Pajević, Živko Ćurčić, Jovica Vasin, Aleksandar Djordjevic

**Affiliations:** 1 University of Novi Sad, Faculty of Sciences, Department of Biology and Ecology, Novi Sad, Serbia; 2 University of Novi Sad, Faculty of Sciences, Department of Chemistry, Biochemistry and Environmental Protection, Novi Sad, Serbia; 3 Institute of Field and Vegetable Crops, Novi Sad, Serbia; University College Dublin, IRELAND

## Abstract

Over the past few years, significant efforts have been made to decrease the effects of drought stress on plant productivity and quality. We propose that fullerenol nanoparticles (FNPs, molecular formula C_60_(OH)_24_) may help alleviate drought stress by serving as an additional intercellular water supply. Specifically, FNPs are able to penetrate plant leaf and root tissues, where they bind water in various cell compartments. This hydroscopic activity suggests that FNPs could be beneficial in plants. The aim of the present study was to analyse the influence of FNPs on sugar beet plants exposed to drought stress. Our results indicate that intracellular water metabolism can be modified by foliar application of FNPs in drought exposed plants. Drought stress induced a significant increase in the compatible osmolyte proline in both the leaves and roots of control plants, but not in FNP treated plants. These results indicate that FNPs could act as intracellular binders of water, creating an additional water reserve, and enabling adaptation to drought stress. Moreover, analysis of plant antioxidant enzyme activities (CAT, APx and GPx), MDA and GSH content indicate that fullerenol foliar application could have some beneficial effect on alleviating oxidative effects of drought stress, depending on the concentration of nanoparticles applied. Although further studies are necessary to elucidate the biochemical impact of FNPs on plants; the present results could directly impact agricultural practice, where available water supplies are often a limiting factor in plant bioproductivity.

## Introduction

Drought is an important environmental factor with a strong negative impact on agriculture. The frequency of heat waves and associated periods of drought are predicted to increase in some parts of Europe [1,2]. In fact, climate change will be one of the main driving forces determining agricultural plant yields, performance and stability [3]. Low water availability is one of the main environmental factors influencing plant growth and yield in many regions of the world [4]. Therefore, it is increasingly important to raise environmental awareness and improve plant drought tolerance in order to sustain crop quality.

A number of engineered nanomaterials are being investigated for use in agriculture for increasing crop productivity and protection [5,6]. Among these, carbon based nanomaterials (CBNMs) have been the focus of several studies in recent years, and have been shown to be helpful in both agriculture and biotechnology [7,8]. The most investigated CBNMs are fullerene (C_60_ and C_70_), fullerenol C_60_(OH)_x_, x = 18–36 and CNTs. Although some studies have reported positive effects on plant growth and development in crop plants associated with application of CBNMs, few studies have addressed how fullerene molecules and their derivates may affect plants [8,9].

For biological applications, the main disadvantage of native fullerene is its insolubility in water. To overcome this, water-soluble fullerene derivatives have been designed and synthesised, which retain many of the unique properties of native fullerene, while enabling application for the desired biological activity. Because of their high solubility in water, these fullerene derivates represent attractive nanoparticles for various biological applications [10–14]. In particular, fullerene and its derivates exhibit strong antioxidative, antimicrobial, cytoprotective, neuroprotective and radioprotective effects in animal organisms [10,15–20].

Kole et al. [21] introduced FNPs (C_60_(OH)_20_) to different plant tissues in bitter melon (*Momordica charantiai*) by seed priming, resulting in increased biomass, fruit yield and phytomedicine content. Some evidence suggests that fullerenol can penetrate different cell membranes and compartments [22]. Fullerenol dissolved in water forms polyanion nanoparticles whose size and charge depend on the experimental conditions (concentration, pH, temperature, presence of co-solvents). Depending on the pH value of surrounding aqueous solution, fullerenol molecules can exist in deprotonated and/or protonated forms. Polyanions of fullerenol C_60_(OH)_n_(O)_m_^-^ (2 ≤ m+n ≤ 44) form intermolecular hydrogen bonds, and hydrogen bonds with water molecules [23–25]. The ability to bind water molecules make these nanoparticles a potential intracellular depot which can be used if osmotic stress occurs. We hypothesise that FNPs can penetrate through different plant leaf and root tissues, where it binds water molecules in different cell compartments, creating an additional intercellular water reserve and helping to alleviate drought stress. This hydroscopic activity suggests that FNPs may be uniquely beneficial in plants. To test this hypothesis, we investigated the influence of foliar application of fullerenol nanoparticles (C_60_(OH)_24_) on sugar beet plants exposed to drought stress.

## Materials and Methods

C_60_ (99.8% purity) was obtained from Sigma-Aldrich. Demineralised water (17.5 MΩ) was prepared in-house. Br_2_, NaOH and C_2_H_5_OH (Ethanol p.a., ACS reagent, reag. ISO, reag. Ph. Eur., 99.9%) were obtained from Sigma-Aldrich and were all analytical grade.

### Synthesis of fullerenol

Fullerenol (C_60_(OH)_24_) was synthesized and characterized from polybromine derivative C_60_Br_24_. Polybromine derivative C_60_Br_24_ was synthesized by reaction of C_60_ in Br_2_ with FeBr_3_ as catalyst according to a published protocol [26]. Fifty (50) mg of C_60_Br_24_ was mixed in 5 cm^3^ of NaOH (pH 10) for 2h at room temperature. After reaction completion, solvent was evaporated at 40°C, and the mixture was repeatedly rinsed 5 times with 10 cm^3^ of 80% ethanol. Residual NaOH and NaBr were removed from an aqueous solution (20 ml) of fullerenol by ion exchange (20g DOWEX MB50 QC121815 R1) and eluted with demineralised water. The resulting FNP solution in water (pH = 6.5) was evaporated under low pressure affording a dark brown powder substance [27].

### Physical determination of Fullerenol (C_60_(OH)_24_)

FTIR: C_60_(OH)_24_ has the following characteristic peaks: 3427, 1627, 1419, 1080 cm^−1^. ^13^C-NMR (D_2_O): singlet peaks at δ 169.47 ppm and multiplet peak at δ 160−110 ppm. MALDI MS (*m*/*z*): 720 (C_60_^+^), 993 (C_60_(OH)_16_^+^), 1043 (C_60_(OH)_19_^+^), 1061 (C_60_(OH)_20_^+^), 1128 (C_60_(OH)_24_^+^). DTG, DTA and TG show two thermal changes: 1) at 120–395°C corresponding to loss of 35.7% mass (23.7 OH groups); and 2) at 430°C, corresponding to loss of 64.3% mass (note: this is the sublimation temperature of C_60_).

Aqueous solutions of FNPs with final concentrations of 700 μmol/L and 70 μmol/L were prepared for further experiments. After preparation and sonication for 15 min, all examined solutions were stored in the dark at room temperature.

### Nanoparticle characterization

Dynamic light scattering (DLS) was used for determination of hydrodynamic size, and electrophoretic light scattering (ELS) for surface charge (zeta potential) measurements of the analysed samples. The first set of DLS and zeta potential analyses were performed 30 minutes after sonication, concurrent with foliar application (treatment time point). We conducted a second set of DLS and zeta potential analyses to determine if standing for 24h is associated with changes in particle size distribution and charge of the samples. Measurements were conducted on a Zetasizer Nano ZS (Malvern Instruments Inc, UK). DLS probes hydrodynamic size, shape, structure, and stability, as well as aggregation or formation of biomolecular complexes [28,29]. All DLS analyses were performed in triplicate, and zeta potential measurements were conducted in duplicate.

Atomic force microscopy (AFM) was used to characterize morphology and measure the primary particle size distribution of FNPs in analysed model solutions. AFM measurements were conducted after 24h of incubation in the dark. Surface topography and phase images were simultaneously acquired by standard AFM tapping using a commercial NanoScience-Team Nanotec GmbH SNC (Solid Nitride Cone) AFM probe, with the tip radius lower than 10 nm. Highly-orientated pyrolytic graphite HOPG was used as a surface. A multimode quadrex SPM equipped with a nanoscope IIIe controller (Veeco Instruments, Inc.), operated under ambient conditions was used. Aqueous solutions of FNP were diluted with demineralised water and added dropwise to HOPGE before drying under ambient conditions.

Transmission Electron Microscopy (TEM) analyses of aqueous FNPs were performed to confirm the structure of the nanoparticles. TEM analyses were conducted on a JEM 1400 microscope with accelerating voltage 120kv, using a horizontal field width of 173.9 nm and magnification 300000 x, 30 min after sonication.

### Plant material and experimental set

Sugar beet seeds (*Beta vulgaris L*. cultivar LARA) were obtained from the Institute of field and vegetable crops, Novi Sad, Serbia. Initially, 720 seeds were sown in 0.6 liter pots containing 500 g of sandy soil. After one month, 200 morphologically uniformed (by size and number of leaves) and healthy plants were selected for further growth. Plants were grown under semi-controlled greenhouse conditions. Plants were irrigated with tap water (drinking water, pH 7.82, CaCO3 75–150 mg/L, EC 425 μS/cm) to maintain optimal soil humidity. Temperatures ranged from 14–30°C (night/day). Illumination was natural, and depended on outdoor light conditions. Foliar application of FNPs solutions was performed after 4 months of plant growth. Plants were than differentially watered according to three water regimes:

- control (60–70% of -RWC);
- drought 1 (20–30% of RWC);
- drought 2 (10–20% of RWC).

Drought 1 water regime was reached after 8 days and drought 2 after 9 days. All physiological analyses were performed 13 days after FNP exposure. Plants were then grown in optimal conditions for an additional 3 months. Fresh weights of leaves and roots were measured 7 months after germination and 3 months after foliar FNP application. Saccharose content was determined in roots. Its estimation was assessed to define whether or not FNP as well as drought treatments had influence on saccharose production in tested plants.

#### Foliar fullerenol exposure

Leaf areas of 200 plants were measured using an ADC leaf area meter (ADC BioScientific Ltd. UK). The average leaf area per plant was 8834±110.00 mm^2^. Foliar application of FNPs was performed using a glass laboratory sprinkler with rubber hand pump. Pump output calibration was performed by spraying square aluminium foil (20 cm diameter) using distilled water in 10 replications. Foil weight was measured with an analytical scale before and after spraying 20 full pump amplitudes. The glass sprinkler was held at approximately 5 cm distance from the target surface. The average mass of water deposited at the foil was 142.93 ± 13.5 mg. To determine dissipation of water outside the leaf area, 10 plants were sprayed using the same glass sprinkler and water volume, with aluminium foil serving as a background collector. Foil weight was determined before and after spraying. Average dissipation of water outside the leaf area was < 2.36 ±1.82%. Proportions were adjusted to ensure that 142 mg of water solution would be applied to 10000 mm^2^ of leaf area. For each individual plant, the volume of solution was modified according to leaf area. To calculate concentrations per leaf mass, leaf mass and leaf area was measured for 10 average plants. On average, 1 mm^2^ of leaf area weighed 0.4243 ± 0.045 mg. Thus, two FNPs solutions were prepared at concentrations of 700 μmol/L and 70 μmol/L in distilled water. The final concentrations of FNP deposition were 0.01 (F1 treatment) and 0.001 (F2 treatment) nmol mm^-2^ per leaf area, which corresponds to 0.02356 and 0.002356 nmol mg^-1^ FNP per leaf mass. Control group of plants were treated only with water (F0 treatment). FNP solutions were sonicated for 10 minutes, 30 minutes prior to application.

#### Soil characterization and field water capacity calculation

Soil was obtained from a forest plantation nursery near Novi Sad, Serbia (45°17’25.8” latitude, 19°54’14.7” longitude), no specific permissions were required for this location which is a joint research plot of a National project grant. The soil used is alkaline, loamy sandy soil, with low humus and total nitrogen content (Tables 1 and 2). Potted soil was analyzed (pH value, CaCO_3_, N, P, K, humus) by methods officially accepted by YSSS [30, 31]. Soil texture was analysed by combined method of sieving and sedimentation [32]. RWC (%) was determined as follows: soil was weighed, dried at 105°C and re-weighed. Finally, the soil was watered to maximum holding capacity using a Kopecky cylinder (100 cm^3^) and re-weighed. Measurements were conducted on the basis of the following formula:

RWC = 100*(Sn-Sd)/Swm-Sd

Sn-weight of sampled soil;

Sd-weight of dried soil;

Swm-weight of soil with 100 % RWC

**Table 1:** Soil chemical properties.

pH		CaCO_3_	Humus	Total N	Al-P_2_O_5_	Al-K_2_O
In KCl	In H_2_O	%	%	%	mg/100g	mg/100g
7.62	8.16	19.12	1.29	0.111	3.0	6.4

**Table 2:** Soil mechanical composition.

Large sand %	Small sand %	Powder %	Clay %	Texture class
0.93	86.35	8.04	4.68	Loamy small sand

Identical soil mass (0.6 kg) was aliquoted to each pot. After 4 months of growth, each individual plant mass was determined and added to the pot mass. Ten average plants were used to obtain the proportion between plant mass and leaf area. Each plant leaf area was measured and total pot mass was calculated for the 180 remaining pots. Based on these calculations, each total pot mass was measured every day to determine the necessary amount of water required for irrigation.

### Physiological analyses

#### Proline determination

Free proline content was determined using the Bates method [33]. Plant material was sampled from 6 plants per each treatment. 1 g of fresh plant material was measured for each of the 6 replicates.

#### Enzymes activity and estimation of MDA and GSH content

All chemicals were purchased from Sigma (St. Louis, USA). Spectrophotometric determinations were performed using a Beckman DU-65 UV/Visible spectrophotometer (Beckman Instruments, INC.) at 25°C, in triplicate. Approximately 10 g of plant material were ground in liquid nitrogen and stored at -80°C. Crude leaf and root extracts were prepared according to the procedure described by Nikolić et al [34]. Briefly, plant material was homogenized on ice in 0.01 mol/L phosphate buffer, pH 7.0, containing 1 mmol/L EDTA and 1% polyvinylpyrrolidone (PVP). After centrifugation at 4°C 10,000 × *g* for 15 minutes, a transparent supernatant was obtained and used for measuring enzymatic activity. Soluble protein content was determined according to Bradford [35] with bovine serum albumin as a standard.

*Ascorbate peroxidase* (AP_X_; EC 1.11.1.11) activity was determined by observing the decrease in absorbance of each reaction mixture at 290 nm, as described by Nakano and Asada [36].

*Catalase* (CAT; EC 1.11.1.6) activity was assayed by dismutation of hydrogen peroxide in a reaction mixture at 240 nm, as described by Beaumont et al [37].

*Guaiacol peroxidase* (GPx; EC 1.11.1.7) activity measurements were carried out spectrophotometrically using guaiacol as electron donor substrate. The increase in absorption as a result of the formation of oxidized product (tetraguaiacol) was measured at 436 nm using an extinction coefficient of 25.6 mmol^-1^ cm^-1^ [38].

*Glutathione S-transferase* (GST; EC 2.5.1.18) activity was measured by observing conjugation of 1-chloro, 2,4-dinitrobenzene (CDNB) with reduced glutathione (GSH), using a protocol modified from Habig et al. [39].

*Malondialdehyde concentration* (MDA) in crude extracts was determined as described by Devasagayam et al. [40]. The MDA equivalents of the samples were calculated using an extinction coefficient of 1.56 × 10^5^ mol^-1^ cm^-1^.

*Reduced glutathione* (GSH) content was evaluated using the method of Kapetanović and Mieyal [41].

#### Photosynthetic rate, transpiration rate and saccharose content

Photosynthetic (μmol of CO_2_ m^-2^ s^-1^) and transpiration rates (mmol of H_2_O m^-2^ s^-1^) were measured by the LCpro+ portable photosynthesis system (ADC BioScientific Ltd.). Measurements were conducted for each treatment, and were conducted on 10 different plants. Three replicates were recorded for each plant. Light conditions were set using the LCpro+ light unit, which emitted photosynthetic active radiation (PAR) at 1000 μmol m^-2^ s^-1^. The air supply unit provided a flow of ambient air to the leaf chamber at a constant rate of 100 μmol s^-1^. Temperature, CO_2_ concentration, and humidity were at ambient levels.

Saccharose content was determined from filtered beet brei clarified with lead acetate [42].

#### Statistical Analysis

Statistical analyses were conducted using Fisher's Multiple Range Test, at a level of significance of p<0.05, using 2-way factor analyses. Values shown are arithmetic means ± standard deviation. Values in each table or figure, followed by the same letter do not differ significantly.

## Results and Discussion

### Particles size distribution profile and topography

It has been shown that nanomaterials with sizes <100 nm have the greatest potential for different applications in biological systems [43, 44]. However, there are numerous applications in which single particles with dimensions of 250 nm also exhibit favourable characteristics [45]. The properties of nanomaterials ranging between 1 nm and 250 nm can be considered to represent a hybrid of quantum effects between individual atoms and molecules, and those of bulk materials [46]. The processes taking place in this 'nano domain' are a result of the physical characteristics of these materials and are directly dependent on size, or 'nano size' [47]. Thus, the size of nanoparticles is a primary feature that determines their characteristics and potential effects: for example size reduction leads to increase in the number of nanoparticles per unit mass [48]. In addition to particle size, another important parameter is the ability to form nanoaggregates. Determining the size of agglomerates/aggregates as well as the degree to which these processes take place is essential for interpretation of their effects in biological systems. The size of nanoparticles (agglomerate/aggregates) is a key determinant for the process of translocation through the cell membrane. Interactions between living systems and nanoparticles are impacted by a synergy of factors: the size, shape and surface modification of nanoparticles; purity, ability to aggregate, surface charge and chemistry [49]. In order to obtain reliable results, it is necessary to more accurately define and characterize the manner and degree of agglomeration in experimental conditions [49, 50].

Data obtained by DLS, zeta potential, AFM and TEM techniques enable detailed, precise analysis of the specific nature of the analysed nanoparticles.

**Fig 1:**
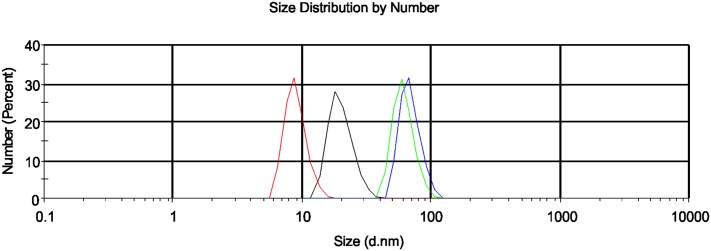
FNP particle size distribution measurements by DLS. DLS size distributions for FNPs at 2 concentrations and time points: 700 μmol/L FNP (black line-30 min, blue line-24h); 70 μmol/L FNP (red line-30 min, green line-24h).

The particle size distribution of aqueous FNP solutions at different time intervals and concentrations are shown in Fig 1. FNP concentrations of 700 μmol/L (30 min-blue line, 24h-green line) and 70 μmol/L (30 min-red line, 24h-green line) were tested. DLS measurements clearly show that the largest number of particles for both FNP concentrations ranged from 6 nm to 39 nm for the first time point (30 min), with maximums at 18 and 9 nm for 700 μmol/L (black line) and 70 μmol/L (red line), respectively. By 24h, sizes ranged from 30 nm to 105 nm, with maximums at 68 nm and 58 nm for 700 μmol/L (blue line) and 70 μmol/L (green line), respectively. Based on these results, differences in FNP concentrations do not appear to significantly affect particle size distribution. After 24 hours, FNPs appear to predominately form stabile clusters with sizes ranging from ~50–70 nm. These data are in close agreement with previously obtained results for FNP particle size distributions, confirming the presence of aggregates with sizes <100 nm [51,52]. Based on the dimensions of the FNPs obtained by DLS, and the theoretically determined radius of the fullerenol molecule (~1.1 nm), it is possible that the FNPs mostly organize in the form of a secondary percolation cluster. Non-aggregated fullerenol molecules were not detected. Based on aggregate dimensions, FNPs appear to have a high propensity to form aggregates. These results are in accordance with the results of Semenov and co-workers [53] and Letenko and co-workers [54].

**Fig 2:**
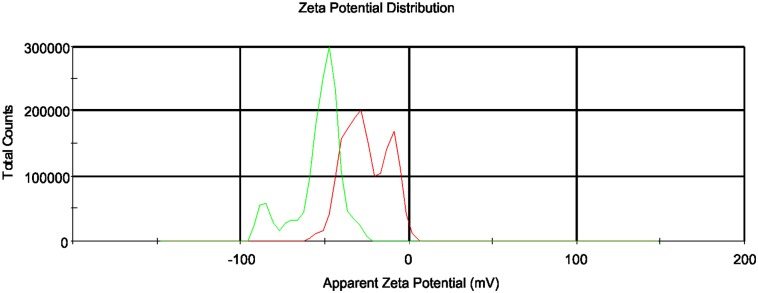
Zeta potential measurements of FNP solutions. FNP concentrations of 700 μmol/L (green line) and 70 μmol/L (red line) were analysed 30 minutes after sonication.

Zeta potential analyses for the same two concentrations of FNP solutions were performed 30 min after sonication (see Fig 2). The mean zeta potential was -31.9 mV for 700 μmol/L FNP (green line) and -53.3 mV for 70 μmol/L FNP (red line), indicating the stability of the FNP systems in both analysed samples. After 24 hours, no significant changes in zeta potential values were evident for either analysed solution. Particles having zeta potential values more positive than +30 mV or more negative than—30 mV are considered to be stable [29,55]. Based on these results, the analysed FNP solutions can be considered to be stable colloidal systems.

**Fig 3:**
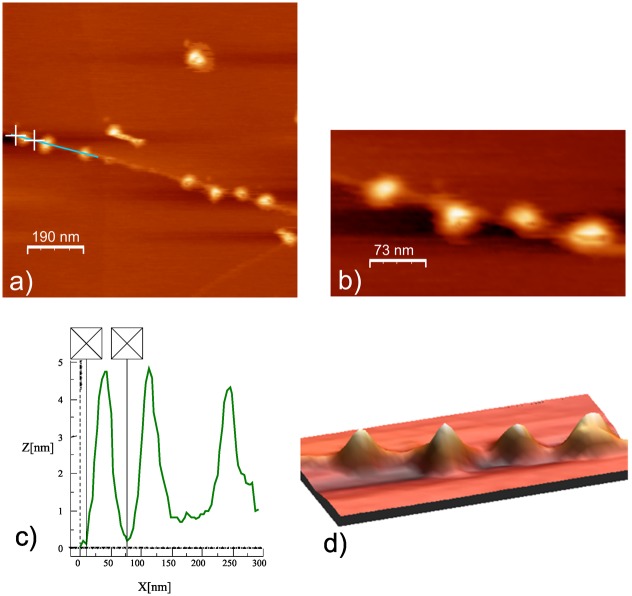
AFM images of FNP solutions after storage at 25°C for 24h. (a) large-scale image, 963 x 963 nm^2^; (b) small scale image 365 x 203 nm^2^; (c) cross-sections of FNPs in water with sizes of 66 nm, 70 nm and 55 nm. The peak represents particle height. The maximum particle height was 4.7 nm; (d) 3D image of FNPs from a small-scale image on the HOPG surface.

Particle size and topography analyses were performed using AFM to obtain detailed structural information for the FNPs. Results of AFM studies with respect to surface morphology and topography are presented in Fig 3. As can be seen, the FNPs samples are in general relatively inhomogeneous. AFM analyses of aqueous FNP solutions suggest aggregate sizes of ~55 nm to 70 nm (Fig 3), consistent with DLS measurements (see Fig 1). Similar to DLS results, AFM confirms the general trend of FNPs aggregates with dimensions less than 100 nm. Coupled with our observed negative zeta potentials for FNP solutions (Fig 2), these AFM results indirectly confirm the expected hydrophilic structure of the FNPs. Consistent with their polarity, most FNP particles were found to be distributed on the HOPG terraces; while the remainder formed hydrophobic interactions with the surrounding non-polar graphite surface, in agreement with literature data [23, 25].

#### Visualization of FNP dispersion

**Fig 4:**
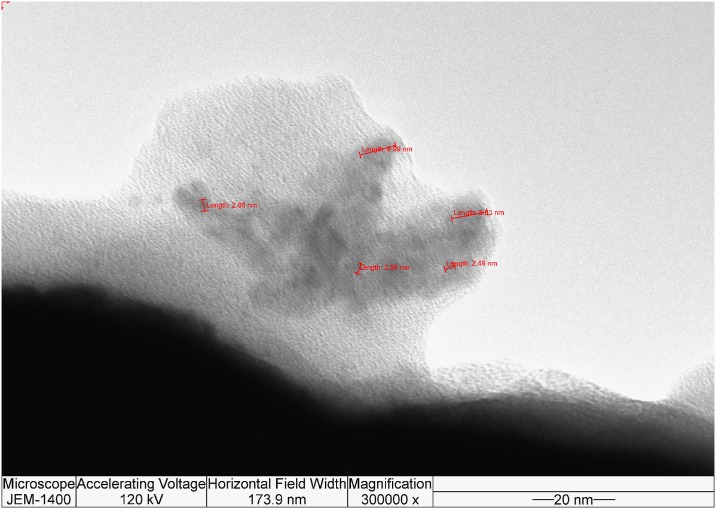
TEM image of FNP solution.

TEM was performed to visualize FNP dispersion in experimental samples. Fig 4 represents TEM analysis of FNPs solutions suggest an inhomogeneous system where the most dominant nanoparticles had sizes <10 nm. Together, DLS, zeta potential, AFM and TEM analyses confirm the stabile hydrophilic structure of FNP aggregates with sizes less than 100 nm.

### Physiological analyses

#### Proline content

**Fig 5:**
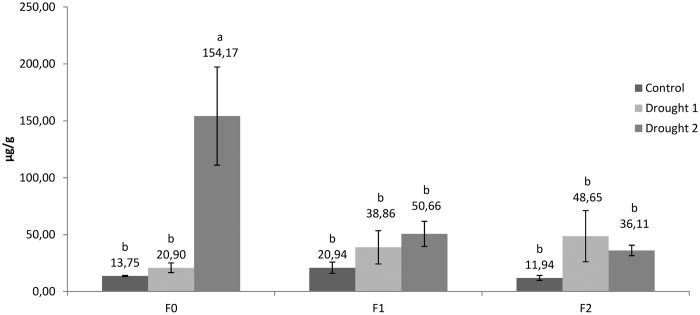
Proline content in sugar beet leaves. Different letters indicate significant differences between treatments according to Fisher’s test (p < 0.05), representing the means of three independent measurements ± SD.

**Fig 6:**
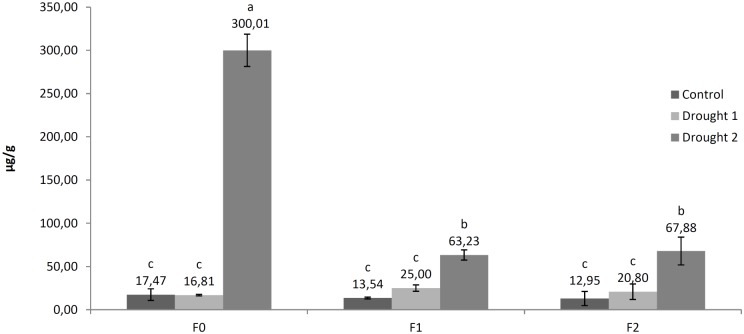
Proline content in roots of sugar beet. Different letter indicate significant differences between treatments according to Fisher’s test (p < 0.05), representing the means of three independent measurements ± SD.

Proline content was determined in leaves and roots (Figs 5 and 6). Moderate drought treatment (drought 1) did not cause significant modifications of proline content in both leaves and roots. However, severe drought treatment (drought 2) for 4 days significantly increased proline content in the leaves of plants not treated with FNPs (154.17 μg/g fresh weight; Fig 5). Proline content in leaves was 11.2 fold higher than that determined in control plants held under an optimal water regime. Accumulation of proline is regarded as an adaptive metabolic acclimation of plants to drought stress. Although proline is regarded as a compatible osmolyte because of its role in osmotic adjustment, it plays other important roles associated with protective activity during drought stress. For example, proline can act as a free radical scavenger, a stabiliser of intracellular structures, macromolecules and membranes, an activator of detoxification pathways, a source of energy, nitrogen and carbon, and a signalling molecule [56,57]. Interestingly, our results strongly suggest that proline is *not* accumulated in plants treated with FNPs, even following exposure to severe drought (drought 2 treatments, 50.66 and 36.11 μg/g fresh weight, respectively), affording speculation that nanoparticle treatment reduces the need for proline accumulation in drought stressed leaves.

Fullerenol can bind large amounts of water, thus acting as a compatible osmolyte able to serve as an additional intracellular supply of water. During foliar application of FNPs, plants were supplied with optimal amounts of water (soil RWC 60–70%), so that sufficient water was able to accumulate in circular layers around the fullerenol core. This water is likely released only when drought stress significantly reduces the cells osmotic potential, so that differences in water potential provide a stronger diffusion force than the hydrogen bonds between water molecules and FNPs. Given this hypothesis, it is tempting to speculate that fullerenol could act as both an osmotic adjuster and stabiliser of intracellular structures. In this case, it is possible that additional increases in proline concentrations would not be necessary under drought stress, since its activity is replaced by the FNPs.

Proline content in roots was significantly increased by 17.2 fold under drought 2 treatment (300.01 μg/g fresh weight) *vs*. controls (17.47 μg/g fresh weight) (Fig 6). Higher proline content was also observed for fullerenol treated plant roots under drought 2 treatment *vs*. control plants.

However, similar to results obtained for proline content in leaves, fullerenol treatment was associated with significant modifications of proline accumulation in roots. In comparison to plants not treated with nanoparticles (F0), fullerenol treatments (F1 and F2) also significantly reduced proline accumulation in roots following drought 2 treatment (300.01, 63.23 and 67.88, respectively). Furthermore, these results suggest that after foliar application, FNP is able to pass through membrane structures to reach the root tissues. Thus, the present study represents indirect evidence that FNPs are mobile in tissues of sugar beet, suggesting that they in general can penetrate through different biomembranes. However, these findings must be supported by direct identification of fullerenol in different plant parts. In aggreement with the present study, fullerenol mobility through plant tissues has been reported in bitter melon (*Momordica charantia*), with a positive correlated increase of plant water content and fruit yield [21]. These authors explain that the most probable cause of increased yield is related to increased plant water content. Similarly, Khodakovskaya et al. [58] determined that increase in germination and growth in tomato seeds treated with CNTs is supported by increased seed water uptake. These authors also observed high mobility of carbon nanoparticles inside plant and seed tissues. In general, CBNMs have been found to be highly mobile in plant tissues, depending on the applied nanoparticle [8,59]. Further biochemical, genomic and proteomic studies are needed to fully understand FNPs mobility and physiological activities in plants.

#### Antioxidant enzyme activities, MDA and reduced glutathione content

**Fig 7:**
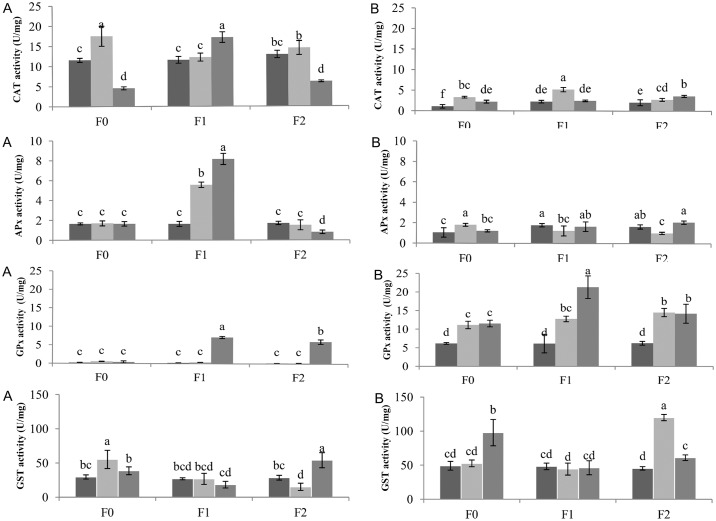
CAT, APx, GPx and GST activities in the leaves (A) and roots (B) of sugar beets after exposure to drought stress. *dark bars–*Control*, light gray bars—*Drought 1*, gray bars—*Drought 2*. Enzymes activities are expressed in units of enzyme activity per milligram of protein. Different letters in the same chart indicate significant differences between treatments according to Fisher’s test (p < 0.05), representing the means of three independent measurements ± SD.

**Fig 8:**
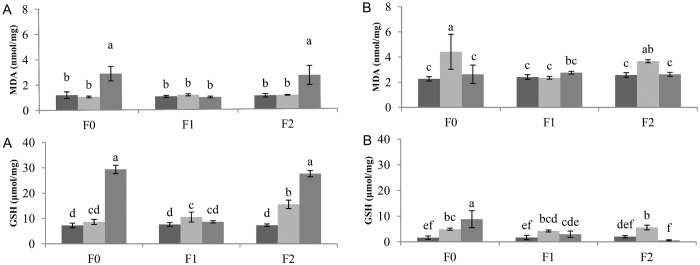
Malondialdehyde (MDA) and reduced glutathione (GSH) content in the leaves (A) and roots (B) of sugar beets after exposure to drought stress. *dark bars–*Control*, light gray bars—*Drought 1*, gray bars—*Drought 2*. MDA and GSH contents are expressed per milligram of protein. Different letters in the same chart indicate significant differences between treatments according to Fisher’s test (p < 0.05), representing the means of three independent measurements ± SD.

Exposure to ROS can result in enhanced antioxidant capacity. According to O’Brien et al. [60], the majority of ROS produced in response to stress conditions is H_2_O_2_. CAT, APx and GPx are well-known enzymes involved in the detoxification of H_2_O_2_ via conversion to water and oxygen. [61,62]. However, there is a lack of data on the effects of FNPs on plant cell oxidative properties. To address this, we determined the dynamic change in activity of these enzymatic systems as a function of FNP concentration and induced drought stress. In general, under control (well-watered) conditions, foliar applications of FNP did not affect the activity of the examined enzymes, or MDA and GSH content (Figs 7 and 8). Both CAT and APx activity were up to 3-fold higher in the leaves of sugar beets vs. roots. CAT activity varied between treatments. Moderate drought clearly has a significant (p<0.05) effect on CAT activity in the F0 treatment, resulting in increased enzyme activity in comparison to F1 and significantly vs. F2 (Fig 7). In contrast, severe drought stress negatively influenced CAT activity in F0 and F2 plants, compared with F1. Ascorbate Px activity significantly increased under water stress conditions at F1 fullerenol concentrations (Fig 7). APx activity was not affected in treatments F0 and F2 in both drought stress conditions in comparison with control. A similar pattern of APx activities was observed in plant roots at both FNP concentrations. A slight decrease of activity was evident under moderate drought treatment, in comparison with other treatments. With respect to H_2_O_2_ scavenging enzyme activity, it appears that both CAT and APx activities are more susceptible to severe drought stress in the leaves of F0 and F2 plants, consistent with our observed decrease in enzymatic activities vs. well-watered and F1 plants. It is possible that the severe drought regime induces more intensive oxidative stress and possible loss of cell turgor, causing eventual failure of plant defence mechanisms. On the contrary, F1 plants had a significant increase in CAT and APx activities, suggesting FNPs treatment may have a positive effect on plant defence mechanisms against drought stress. In favour of this is the fact that FNPs treatment is associated with beneficial antioxidative properties, in agreement with reports from animal cells [63]. It is interesting to note that not all nanomaterials have a positive effect on plant antioxidant defence systems. According to Zhao et al. [64] CAT and APx activities in corn shoots were highly susceptible to application of CeO_2_ nanoparticles, implying that high levels of nanoparticles may actually lead to inhibition of enzyme activities. In another study, Faisal et al. [65] reported *elevated* CAT and SOD activities in tomato plants with increasing NiO nanoparticles concentrations, leading to oxidative stress and mitochondrial dysfunction. In contrast, Song et al. [66] did not find any physiological differences in total antioxidant capacity (TAC) or superoxide dismutase activity (SOD) in *L*. *sativa* i *B*. *campestris* plants following nano-TiO_2_ treatments.

While CAT and APx activity was mainly found in the leaves (with very low activity observed in plant roots), GPx activity was actually enhanced in plant roots. This reduced GPx enzyme activity in plant leaves may be due to differential distribution, expression and/or activity of enzyme isozymes [67–69]. The highest GPx activity was observed in the roots of sugar beets subjected to severe stress and foliar FNPs application at the F1 concentration: up to 21.31 U mg of protein^-1^ (Fig 7). All activities observed in FNPs treatment groups were statistically different from untreated controls. GST activity was also higher in the roots of tested plants, similar to results obtained for GPx. While there were no significant differences between GST activities in the roots of F1 plants subjected to water stress, control treatments displayed a significant response in plant roots following severe drought stress (Fig 7). Similary, high GST activity was measured in the roots of F2 treated plants under moderate drought stress conditions. GST activities in F1 treated plant leaves under different water regimes did not show significant differences. Higher GST activity was found in F0 (severe drought) and F2 plants (moderate drought), in strong agreement with measured GSH and MDA levels. In fact, a complex array of compounds are produced during lipid peroxidation which may be considered to be reactive electrophile species “RES” [70]. The biological activity of RES can be regulated by conjugation to glutathione. Although such conjugation can occur spontaneously, it is accelerated by GST activity [71]. Elevated RES accumulation, followed by conjugation can markedly deplete glutathione pools [71,72].

As key antioxidants, ascorbate and glutathione can be used as biochemical markers of general cell redox state [73]. For example, leaf glutathione status is clearly influenced by intracellular H_2_O_2_ availability [74]. We have noticed that accumulation of GSH was 3-fold higher in treatments with decreased CAT and APx activities (F0 and F2 plants exposed to severe drought). The close link between increased intracellular H_2_O_2_ and changes in glutathione status was underscored in a CAT-deficient *Arabidopsis* mutant cat2 [75,76]. Furthermore, activation of GSH synthesis and accumulation of glutathione is a general feature of enhanced oxidation in the cytosol [77]. Besides antioxidant enzymes activities, we investigated whether induced water shortage was associated with oxidative stress and damage to membrane lipids and GSH content. Lipid peroxidation levels (measured as MDA content) and GSH content in the leaves and roots of sugar beets are shown in Fig 8. MDA content was ~2-fold higher in the roots of sugar beets. Significant differences were only evident for the moderate drought stress treatment for F0 and F2 plants. Similarly, in the leaves of sugar beet plants, higher MDA levels were found in F0 and F2 plants subjected to severe water deficit (Fig 8). MDA content in F1 plants was similar to controls in both the roots and leaves. Water deficiency significantly (p<0.05) increased GSH levels in the leaves of F0 and F2 (29.4 and 27.7 U mg protein^-1^, respectively) plants exposed to severe drought stress. Non-significant increases in GSH content in leaves were observed for F1 under both water deficit regimes. GSH content was 2 to 4-fold smaller in roots vs. the leaves of sugar beet plants. However, significant GST increases were found for all three FNPs concentrations and water deficit treatments.

MDA represents a well-studied, reactive aldehyde lipid peroxidation product [71] which is CAT insensitive [60]. Whether H_2_O_2_ will act as a signalling molecule or cause damage depends on the delicate equilibrium between H_2_O_2_ production and cell scavenging mechanisms [64]. In contrast to F1 plants, F0 and F2 plants subjected to severe drought displayed increased levels of membrane lipid peroxidation in leaves. Zhao et al. [64] did not find significant differences in lipid peroxidation levels among applied treatments of CeO_2_ nanoparticles in the roots or shoots of corn plants. However, Borsani et al. [78] reported that induced water stress resulted in a −0.46 MPa decrease in hydric potential, associated with membrane lipid peroxidation in the leaves of *Lotus corniculatus* plants. Our observed differences in peroxidation levels possibly suggest a beneficial effect associated with FNPs treatment on plant metabolism under altered soil water conditions.

#### Photosynthetic and transpiration rates

Photosynthetic and transpiration rates are shown in Table 3. Photosynthetic rates were not affected by FNPs treatment. Drought 2 treatment significantly reduced CO_2_ assimilation in comparison to control treatment, but these reductions were not significantly modified by FNP exposure. Drought 2 treatment also reduced transpiration rates in comparison with the control (optimal) water regime. Similar relationships were found between transpiration and photosynthetic rates.

**Table 3:** Photosynthetic and transpiration rate of treated plants.

	Control			Drought 1			Drought 2		
	F0	F1	F2	F0	F1	F2	F0	F1	F2
Photosynthetic rate(μmol CO_2_ m^-2^ s^-1^)	4.94±0.58 a	4.99±0.59 a	4.83±0.56 ab	4.85±0.65 ab	4.46±0.53 ab	4.18±0.55 ab	2.93±0.60 b	2.91±0.61 b	2.96±0.58 b
Transpiration rate(mmol H_2_Om^-2^ s^-1^)	0.96±0.15 a	1.00±0.12 a	0.94±0.15 a	0.88±0.15 ab	0.86±0.13 ab	0.85±0.16 ab	0.60±0.13 b	0.59±0.13 b	0.68±0.12 ab

^a,b^ values followed by the same letter are not significantly different according to Fisher’s test (p < 0.05)

Decreased photosynthetic rates under drought stress conditions may be due to disturbance of specific biochemical processes, such as decreased ribulose bisphosphate carboxylase (Rubisco) activity [79]. Carbon balance is disturbed by reduced intercellular CO_2_ concentrations as a result of stomatal closure and CO_2_ diffusion limitations under reduced free water conditions [4]. Although our results indicate that FNPs may modify osmotic adjustment mechanisms, significant changes in photosynthetic and transpiration rates were not observed. If osmotic adjustment is modified by FNPs, it could be beneficial during severe water deficits, especially if it enables stomata to remain partially open; thus allowing additional CO_2_ uptake and water transpiration. Values for transpiration rates for F2 were higher compared to F0 under drought 2 treatment, suggesting additional available water. However these differences were not statistically significant and not in correlation with photosynthetic rate measurements.

#### Fresh weight and saccharose content

Fresh weights of leaves and roots, and saccharose content are shown in Table 4. These measurements were conducted 3 months after drought treatments and foliar nanoparticle exposure. During these 3 months all plants were grown under optimal watering conditions. Both leaf and root mass were not significantly affected by FNP treatment. Differences between the fresh weight of leaves and roots were not statistically significant, mostly because of the high standard deviation associated with these measurements.

**Table 4:** Fresh weight of leaves and roots and saccharose content in roots.

	Control			Drought 1			Drought 2		
	F0	F1	F2	F0	F1	F2	F0	F1	F2
Leaf mass (g)	6.2±1,2 a	6.0±1.8 a	5.8±0.9 a	5.3±0.9 a	5.6±0.8 a	5.6±0.8 a	5.5±0.8 a	5.7±0.7 a	5.2±0.8 a
Root mass (g)	6.1±1.4 a	5.7±1.6 a	5.2±0.7 a	5.4±1.2 a	5.1±0.8 a	5.5±1.1 a	5.6±1.6 a	5.5±0.9 a	5.2±1.5 a
Saccharose(%)	15.5±1.1 ab	17.6±1.1 a	16.5±0.9 ab	15.8±0.8 ab	16.5±0.6 ab	16.8±0.9 ab	14.3±0.7 b	15.5±1.0 ab	15.5±0.9 ab

Measurements were conducted 3 months after foliar application of FNPs.

^a,b^ values followed by the same letter are not significantly different according to Fisher’s test (p < 0.05)

Although average values of saccharose content in roots were higher in fullerenol treated plants vs. untreated controls; these saccharose content modifications were mostly not supported by statistical analysis. A significant decrease in saccharose content in drought 2 treated F0 plants (14.3%) was observed in comparison to control plants treated with F1 FNPs concentrations (17.6%). However, this trend suggests that FNPs treatment is not associated with statistically significant modifications of saccharose content in plant roots.

The short duration of our simulated drought (4 days) could be a significant factor preventing observation of the effect of FNPs on growth and saccharose content. The real impact of FNP on yield quantity and quality should be analysed in field tests using technologically usable plants (matured plants ready for harvesting), for yield and saccharose content analyses. Kole et al. [21] treated bitter melon (*Momordica charantia*) seed with five nanomolar concentrations of fullerenol C_60_(OH)_20_; and observed an up to 54% increase in biomass yield, 24% increase in water content and 128% increase in fruit yield. They also reported increased active anticancer and antidiabetic phytomedicine molecules in nanoparticle treated fruits [21]. They determined that selection of the optimal concentration of nanoparticle is important for targeted phyisological effects on plants and analogue agroeconomic traits. CNTs also had a significant stimulative impact on vegetative biomass of germinated seedlings in Tomato seeds. Some of the proposed mechanisms of these effects are increased water uptake by seed coat penetration and regulation of water channel (aquaporin) gating [58]. Germination was also promoted by CNTs in wheat [80]. Enhanced germination, growth and active molecule concentrations in plants exposed to different CBNMs are mostly explained by their ability to enhance water uptake. The germination of tomato plants was dramatically higher for seeds germinated in medium containing 10–40 mg/ml of CNTs, which supported better water uptake inside the seeds [58]. The germination percentage and germination index of wheat increased significantly in the presence of CNTs in concentrations ranging from 320 mg/ml to 2560 mg/ml [80]. Authors suggest it is posiblle that nanotubes penetrated the soft seed coat of wheat thus increasing its availability for water uptake. Corn seedlings exposed to various levels of multi wall CNTs in agar gel had improved growth and water uptake [81]. Number of analyses also showed some beneficial effect of carbon nanotube suspensions on germination and growth of various agriculture plant species such as Allium cepa Brassica juncea, Brassica napus, Cucumis sativus Daucus carota, Lolium perenne, Raphanus sativus and Satureja khuzestanica [82–85]. However, further proteomic and genomic analyses are required to further elucidate the precise biochemical mechanisms of such activity in plants.

Little is still known about the potential hazards of nanomaterial applications in agriculture, although some reports have recently emerged [5,86,87]. Similarly, although nanomolar application of fullerenol in animal tissues appears to be harmless, [88–90] future studies should address whether FNPs are safe for use in both agriculture and other non-food plant growth systems. Up to date there are no known references about negative impact of FNPs to plants, their metabolism and survival. Results of our research emphasize the importance of further studies in order to elucidate the biochemical impact of FNPs on plants. These findings could be relevant for agricultural, horticultural and bioenergy industries, especially considering that water limitations are an increasingly important factor in future plant bioproductivity.

## Abbreviations

FNPs: fullerenol nanoparticles
CBNMs: carbon based nanomaterials
CNTs: carbon nanotubes
DLS: dynamic light scattering
ELS: electrophoretic light scattering
AFM: atomic force microscopy
TEM: transmission electron microscopy
RWC: relative water capacity
ROS: reactive oxygen species
CAT: catalase
GST: gluthathione-s-transferase
GPx: guaiacol-peroxidase
APx: ascorbate-peroxidase
MDA: malondialdehyde concentration
GSH: reduced glutathione content

## References

[1] Schär C, Vidale PL, Lüthi D, Frei C, Häberli C, Liniger MA, et al The role of increasing temperature variability in European summer heat waves. Nature. 2004;427:332–6. 10.1038/nature02300 14716318

[2] Spinoni J, Naumann G, Vogt J, Barbosa P. European drought climatologies and trends based on a multi-insicator approach. Glob Planet Change. 2015;127:50–7.

[3] Kertész A, Madarász B. Conservation agriculture in Europe. Int Soil Water Conserv Res. 2014;1(2):91–6.

[4] Chaves MM, Maroco JP, Pereira JS. Understanding plant responses to drought—from genes to the whole plant. Funct. Plant Biol. 2003;30:239–64. 10.1071/FP02076 32689007

[5] Bouwmeester H, Dekkers S, Noordam MY, Hagens WI, Bulder AS, de Heer C, et al Review of health safety aspects of nanotechnologies in food production. Regul. Toxicol. Pharmacol. 2009;53:52–62. 10.1016/j.yrtph.2008.10.008 19027049

[6] Khot LR, Sankaran S, Maja JM, Ehasni R, Schuster EW. Applications of nanomaterials in agricultural production and crop protection: A review. Crop Prot. 2012;35:64–70.

[7] Nair R, Varghese SH, Nair BG, Maekawa T, Yoshida Y, Kumar DS. Nanoparticulate material delivery to plants. Plant Sci. 2010;179:154–63.

[8] Husen A, Siddiqi KS. Carbon and fullerne nanomaterials in plant system. J. Nanobiotechnology 2014;12–6. 24766786 10.1186/1477-3155-12-16 PMC4014205

[9] Arruda SCC, Silva ALD, Galazzi RM, Azevedo RA, Arruda MAZ. Nanoparticles applied to plant science: A review. Talanta 2015;131:693–705. 10.1016/j.talanta.2014.08.050 25281161

[10] Da Ros T. Twenty years of Promises: Fullerene in Medicinal Chemistry In: Cataldo F, Da Ros T, editors. Medicinal Chemistry and Pharmacological Potential of Fullerenes and Carbon Nanotubes. New York, NY, United States: Springer-Verlag; 2008 pp. 1–23.

[11] Nielsen GD, Roursgaard M, Jensen KA, Poulsen SS, Larsen ST. In vivo biology and toxicology of fullerenes and their derivatives. Basic Clin Pharmacol Toxicol. 2008;103:197–208. 10.1111/j.1742-7843.2008.00266.x 18684229

[12] Partha R, Conyers JL. Biomedical applications of functionalized fullerene-based nanomaterials. Int J Nanomed. 2009;4:261–75. PMC2789438 20011243

[13] Ma H, Liang XJ. Fullerenes as unique nanopharmaceuticals for disease treatment. Sci China Chem. 2010;53:2233–40.

[14] Prylutska S, Bilyy R, Overchuk M, Bychko A, Andreichenko K, Stoika R, et al Water-soluble pristine fullerenes C60 increase the specific conductivity and capacity of lipid model membrane and form the channels in cellular plasma membrane. J Biomedical Nanotechnol. 2012;8:522–7. 10.1166/jbn.2012.1404 22764423

[15] Djordjević A, Bogdanović G. Fullerenol-a new nanopharmaceutic? Archive of Oncology 2008;16:42–5.

[16] Bakry R, Vallant RM, Najam-ul-Haq M, Rainer M, Szabo Z, Huck CW, et al Medicinal applications of fullerenes, Int. J. nanomed. 2007;2:639–49. PMC2676811 18203430

[17] Bogdanović V, Stankov K, Ičević I, Žikić D, Nikolić A, Šolajić S, et al Fullerenol C_60_(OH)_24_ effects on antioxidative enzymes activity in irradiated human erythroleukemia cell line. J. Radiat. Res. 2008;49:321–7. 18285660 10.1269/jrr.07092

[18] Injac R, Perse M, Cerne M, Potočnik N, Radić N, Govedarica B, et al Protective effects of fullerenol C_60_(OH)_24_ against doxorubicin-induced cardiotoxicity and hepatotoxicity in rats with colorectal cancer. Biomaterials. 2009;30:1184–96. 10.1016/j.biomaterials.2008.10.060 19046599

[19] Stankov K, Borišev I, Kojić V, Rutonjski L, Bogdanović G, Đorđević A. Modification of antioxidative and antiapoptotic genes expression in irradiated K562 cells upon fullerenol C_60_(OH)_24_ nanoparticle treatment. J Nanosci Nanotechnol. 2013;13:105–113. 23646704 10.1166/jnn.2013.6847

[20] Djordjević A, Srdjenović B, Seke M, Petrović D, Injac R, Mrđanović J. Review of Synthesis and Antioxidant Potential of Fullerenol Nanoparticles. J. Nanomater. 2015; 10.1155/2015/567073

[21] Kole C, Kole P, Randunu KM, Choudhary P, Podila R, Ke PC, et al Nanobiotechnology can boost crop production and quality: first evidence from increased plant biomass, fruit yield and phytomedicine content in bitter melon (*Momordica charantia*). BMC Biotechnol. 2013;13–37. 23622112 10.1186/1472-6750-13-37 PMC3644254

[22] Foley S, Crowley C, Smaihi M, Bonfils C, Erlanger BF, Seta P, et al Cellular localization of a water-soluble fullerene derivative, *Biochem*. Bioph. Res. *Co*. 2002;294:116–9. 10.1016/S0006-291X(02)00445-X 12054749

[23] Husebo LO, Sitharaman B, Furukawa K, Kato T, Wilson LJ. Fullerenols revisited as stable radical anions, J. Am. Chem. Soc. 2004;126:12055–64. 10.1021/ja047593o 15382940

[24] Vileno B, Marcoux PR, Lekka M, Sienkiewicz A, Fehér T, Forró L. Spectroscopic and Photophysical Properties of a Highly Deivatized C_60_ Fullerol. Adv *Funct* Mater. 2006;16:120–08.

[25] Brant JA, Labille J, Bottero J, Wiesner MR. Characterizing the impact of preparation method on fullerene cluster structure and chemistry. Langmuir. 2006;22:3878–85. 10.1021/la053293o 16584270

[26] Djordjević A, Vojinović-Miloradov M, Petranović N, Devečerski A, Lazar D, Ribar B. Catalytic preparation and characterization of C_60_Br_24_, Fullerene Sci. Techn. 1998;6:689–94.

[27] Mirkov S, Djordjević A, Andric N, Andric S, Kostic T, Bogdanovic G, et al Nitric oxide-scavenging activity of polyhydroxylated fullerenol C_60_(OH)_24_. Nitric Oxide. 2004;11:201–7. 10.1016/j.niox.2004.08.003 15491853

[28] Inagaki S, Ghirlando R, Grisshammer R. Biophysical characterization of membrane proteins in nanodiscs. Methods. 2013;59:287–300. 10.1016/j.ymeth.2012.11.006 23219517 PMC3608844

[29] Sapsford KE, Tyner KM, Dair BJ, Deschamps JR, Medintz IL. Analyzing nanomaterial bioconjugates: a review of current and emerging purification and characterization techniques. Anal Chem. 2011;83:4453–88. 10.1021/ac200853a 21545140

[30] YSSS—Yugoslav Soil Science Society (1966): Priručnik za ispitivanje zemljišta Knjiga I. Hemijske metode ispitivanja zemljišta. Beograd pp. 124–30.

[31] YSSS—Yugoslav Soil Science Society (1997): Priručnik za ispitivanje zemljišta Knjiga V. Metode istraživanja fizičkih svojstava zemljišta. Beograd pp. 17–32.

[32] Tommerup EC. The Field Description of the Physical Properties of Soils, First Commission of Commission I—Soil Physics—Of the International Society of Soil Science, International Society of Soil Science, Versailles, France. 1934. pp. 155–58.

[33] Bates IS, Waldrn RP, Teare ID. Rapid determination of free proline for water stress. Plant Soil. 1973;39:205–7.

[34] Nikolić N, Kojić D, Pilipović A, Pajević S, Krstić B, Borišev M, et al Responses of hybrid poplar to cadmium stress: photosynthetic characteristics, cadmium and proline accumulation, and antioxidant enzyme activity. Acta Biol Cracov. 2008;502:95–103.

[35] Bradford MM. A rapid and sensitive for the quantitation of microgram quantitites of protein utilizing the principle of protein-dye binding. Anal. Biochem. 1976;72:248–54. 942051 10.1016/0003-2697(76)90527-3

[36] Nakano Y, Asada K. Hydrogen peroxide is scavenged by ascorbate-specific peroxidase in spinach chloroplasts. Plant Cell Physiol. 1981;22:867–80.

[37] Beaumont F, Jouve HM, Gagnon J, Gaillard J, Pelmont J. Purification and properties of a catalase from potato tubers(*Solanum tuberosum*). Plant Sci. 1990;72:19–26.

[38] Simon LM, Fatrai Z, Jonas DE, Matkovics B. Study of metabolism enzymes during the development of *Phaseolus vulgaris*. Biochem Physiol Pflanzen. 1974;166:387–92.

[39] Habig WH, Pabst MJ, Jakoby WB. Glutathione S-transferases. The first enzymatic step in mercapturic acid formation. J. Biol. Chem. 1974;249:7130–9. 4436300

[40] Devasagayam TPA, Boloor KK, Ramasarma T. Methods for estimating lipid peroxidation: an analysis of merits and demerits. Indian J. Biochem. Biophys. 2003;40:300–8. 22900323

[41] Kapetanović IM, Mieyal II. Inhibition of Acetaminophen Induced Hepatotoxicity by Phenacetin and Its Alkoxy Analogs. J. Pharmacol. Exp. Ther. 1979;209:25–30. 430376

[42] ICUMSA, Method GS 6–1, The Determination of the Polarisation of Sugar Beet by the Macerator or Cold Aqueous Digestion Methodusing Lead Acetate as Clarifying Agent. In: ICUMSA Methods Book, 1994.

[43] Lobatto ME, Fuster V, Fayad ZA, Mulder WJ. Perspectives and opportunities for nanomedicine in the management of atherosclerosis. Nature Reviews Drug Discovery. 2011;10(11):835–52. 10.1038/nrd3578 22015921 PMC3623275

[44] Alexis F, Pridgen E, Molnar LK, Farokhzad OC. Factors affecting the clearance and biodistribution of polymeric nanoparticles. Molecular pharmaceutics. 2008;5(4):505–15. 10.1021/mp800051m 18672949 PMC2663893

[45] Papazoglou ES, Parthasarathy A. BioNanotechnology. Synthesis Lectures on Biomedical Engineering. 2007;2(1):1–139.

[46] Singh B. Textbook of Animal Biotechnology: The Energy and Resources Institute (TERI); 2012 620 p.

[47] Pitkethly M. Nanomaterials—the driving force. Materials today. 2004:7(12, Supplement):20–9.

[48] Jiang W, Kim BY, Rutka JT, Chan WC. Nanoparticle-mediated cellular response is size-dependent. Nat Nanotechnol. 2008;3:145–50. 10.1038/nnano.2008.30 18654486

[49] Nel AE, Madler L, Velegol D, Xia T, Hoek EMV, Somasundaran P, et al Understanding biophysicochemical interactions at the nano-bio interface, Nature Materials. 2009;8:543–57. 10.1038/nmat2442 19525947

[50] Oberdorster G. Safety assessment for nanotechnology and nanomedicine:concepts of nanotoxicology. J Intern Med. 2009;267:89–105. 10.1111/j.1365-2796.2009.02187.x 20059646

[51] Mrdjanović J, Šolajić S, Bogdanović V, Djordjević A, Bogdanović G, Injac R, et al Effects of fullerenol nanoparticles C_60_(OH)_24_ on micronuclei and chromosomal aberrations’ frequency in peripheral blood lymphocytes. Dig J Nanomat Bios. 2012;7:673–86.

[52] Slavić M, Djordjević A, Radojičić R, Milovanović S, Oreščanin-Dušić Z, Rakočević Z, et al Fullerenol C_60_(OH)_24_ nanoparticles decrease relaxing effects of dimethyl sulfoxide on rat uterus spontaneous contraction, J Nanopart Res. 2013;15: 1650–58.

[53] Semenov KN, Charykov NA, Keskinov VA. Fullerenol Synthesis and Identification. Properties of the Fullerenol Water Solutions, J Chem. Eng. Data 2011;56:230–9.

[54] Letenko DG, Charykov NA, Nikitin VA, Semenov KN, Matuzenko MY, Keskinov VA, et al Study of Aqueous Solutions of Fullerenol-d by the Dynamic Light Scattering Method. Russ J Appl Chem+. 2011;84:50–3.

[55] Clogston J, Patri A. Zeta potential measurement In: McNeil SE, editors. Characterization of nanoparticles intended for drug delivery. Totowa, NY, United States: Humana Press; 2011 p. 63–70.

[56] Hayat S, Hayat Q, Alyemeni MN, Wani AS, Pichtel J, Ahmad A. Role of proline under changing environments. Plant Signal. Behav. 2012;7(11):1456–66. 10.4161/psb.21949 22951402 PMC3548871

[57] Hossain MA, Hoque MA, Burrit DJ, Fukita M. Proline protects plants against abiotic oxidative stress: Biochemical and molecular mechanisms In: Parvais A. editors. Oxidative Damage to plants, Antioxidant Networks and Signaling. USA: Academic press, Elsevier; 2014 pp. 477–522.

[58] Khodakovskaya M, Dervishi E, Mahmood M, Yang X, Li Z, Fumiya W, et al Carbon nanotubes are able to penetrate plant seed coat and dramatically affect seed germination and plant growth. ACS Nano. 2009;3:3221–7. 10.1021/nn900887m 19772305

[59] Lin S, Reppert J, Hu Q, Hudson JS, Reid ML, Ratnikova TA, et al Uptake, tranlocation, and transmition of carbon nanomaterials in rice plants. Small 2009; (5)10:1128–32. 10.1002/smll.200801556 19235197

[60] O’Brien JA, Daudi A, Butt VS, Bolwell GP. Reactive oxygen species and their role in plant defence and cell wall metabolism. Planta 2012;236:765–79. 10.1007/s00425-012-1696-9 22767200

[61] Shen CX, Zhang QF, Li J, Bi FC, Yao N. Induction of programmed cell death in Arabidopsis and rice by single-wall carbon nanotubes. Am J Bot. 2010;97:1602–9. 10.3732/ajb.1000073 21616795

[62] Gill SS, Tuteja N. Reactive oxygen species and antioxidant machinery in abiotic stress tolerance in crop plants, Plant Physiol. Biochem. 2010;48:909–30. 10.1016/j.plaphy.2010.08.016 20870416

[63] Dugan LL, Lovett EG, Quick KL, Lotharius J, Lin TT, O'Malley KL. Fullerene-based antioxidants and neurodegenerative disorders, Parkinsonism Relat. D. 2001;7:243–6. 10.1016/s1353-8020(00)00064-x 11331193

[64] Zhao L, Peng B, Hernandez-Viezcas J, Rico C, Sun Y, Peralta-Videa J, et al Stress response and tolerance of Zea mays to CeO_2_ nanoparticles: cross talk among H2O2, heat shock protein, and lipid peroxidation. ACS Nano. 2012;6:9615–22. 10.1021/nn302975u 23050848 PMC4326050

[65] Faisal M, Saquib Q, Alatar A, Al-Khedhairy A, Hegazy A, Musarrat J. Phytotoxic hazards of NiO-nanoparticles in tomato: A study on mechanism of cell death. J. Hazard. Mater. 2013;250–251:318–32. 10.1016/j.jhazmat.2013.01.063 23474406

[66] Song U, Shin M, Lee G, Roh J, Kim Y, Lee E. Functional Analysis of TiO_2_ Nanoparticle Toxicity in Three Plant Species. Biol Trace Elem Res. 2013;155:93–103. 10.1007/s12011-013-9765-x 23900645

[67] Yoshimura K, Yabuta Y, Ishikawa T, Shigeoka S. Expression of ascorbate peroxidase isoenzymes in response to oxidative stresses. Plant Physiol. 2000;123:223–33. 10806239 10.1104/pp.123.1.223 PMC58996

[68] Nikravesh F, Khavari-Nejad R, Rahimian H, Fahimi H. Antioxidant enzyme activities and isozyme pattern in hairy roots and regenerated tobacco plants. Russ J Plant Physiol. 2012;59:648–55.

[69] Weng M, Cui L, Fenling L, Zhang M, Shan L, Yang S, et al Effects of drought stress on antioxidant enzymes of different wheat genotypes. Pak. J. Bot. 2015;47(1):49–56.

[70] Farmer EE, Mueller MJ. ROS-mediated lipid peroxidation and RES-activated signaling. Annu. Rev. Plant Biol. 2013;64:429–50. 10.1146/annurev-arplant-050312-120132 23451784

[71] Noctor G, Lelarge-Trouverie C, Mhamdi A. The metabolomics of oxidative stress. Phytochemistry. 2015;112:33–53. 10.1016/j.phytochem.2014.09.002 25306398

[72] Davoine C, Falletti O, Douki T, Iacazio G, Ennar N, Montillet JL, et al Adducts of oxylipin electrophiles to glutathione reflect a 13 specificity of the downstream lipoxygenase pathway in the tobacco hypersensitive response. Plant Physiol. 2006;140:1484–93. 10.1104/pp.105.074690 16500992 PMC1435824

[73] Noctor G, Mhamdi A, Foyer C. The roles of reactive oxygen metabolism in drought: not so cut and dried. Plant Physiol. 2014, 164, 1636–1648. 10.1104/pp.113.233478 24715539 PMC3982730

[74] Mhamdi A, Queval G, Chaouch S, Vanderauwera S, Van Breusegem F, Noctor G. Catalase function in plants: a focus on Arabidopsis mutants as stress-mimic models. J Exp Bot. 2010;61:4197–20. 10.1093/jxb/erq282 20876333

[75] Queval G, Issakidis-Bourguet E, Hoeberichts FA, Vandorpe M, Gakière B, Vanacker H, et al Conditional oxidative stress responses in the Arabidopsis photorespiratory mutant cat2 demonstrate that redox state is a key modulator of day length-dependent gene expression and define photoperiod as a crucial factor in the regulation of H_2_O_2_-induced cell death. Plant J. 2007;52:640–57. 10.1111/j.1365-313X.2007.03263.x 17877712

[76] Queval G, Jaillard D, Zechmann B, Noctor G. Increased intracellular H_2_O_2_ availability preferentially drives glutathione accumulation in vacuoles and chloroplasts. Plant Cell Environ. 2011;34:21–32. 10.1111/j.1365-3040.2010.02222.x 20807372

[77] Foyer CH, Noctor G. Oxidant and antioxidant signalling in plants: a re-evaluation of the concept of oxidative stress in a physiological context. Plant Cell Environ. 2005;28:1056–71.

[78] Borsani O, Díaz P, Agius MF, Valpuesta V, Monza J. Water stress generates an oxidative stress through the induction of a specific Cu/Zn superoxide dismutase in *Lotus corniculatus* leaves. Plant Sci. 2001;161:757–63.

[79] Delfine S, Alvino A, Zacchini M, Loreto F. Consequences of salt stress on CO_2_ diffusion, Rubisco characteristics and anatomy of spinach leaves. Aust. J. Plant Physiol. 1998;25:395–402.

[80] Miralles P, Johnson E, Church TL, Harris AT. Multiwalled carbon nanotubes in alfalfa and wheat: toxicology and uptake. J R Soc. 2014;9:3514–27. 10.1098/rsif.2012.0535 PMC3481593 22977097

[81] Tiwari DK, Dasgupta-Schubert N, Villasenor Cendejas N, Villegas J, Carreto Montoya L, Borjas Garcia SE. Interfacing carbon nanotubes (CNT) with plants: enhancement of growth, water and ionic nutrient uptake in maize (*Zea mays*) and implications for nanoagriculture. Appl Nanosci. 2013; 5:577–91.

[82] Ghorbanpour M, Hadian J. Multi-walled carbon nanotubes stimulate callus induction, secondary metabolites biosynthesis and antioxidant capacity in medicinal plant *Satureja khuzestanica* grown in vitro. Carbon. 2015;94:749–59.

[83] Villagarcia H, Dervishi E, de-Silva K, Biris AS, Khodakovskaya MV. Surface chemistry of carbon nanotubes impacts the growth and expression of water channel protein in tomato plants. Small. 2012;8:2328–34. 10.1002/smll.201102661 22514121

[84] Mondal A, Basu R, Das S, Nandy P. Beneficial role of carbon nanotubes on mustard plant growth: an agricultural prospect. Nanopart Res. 2011;13:4519–28.

[85] Canas JE, Long M, Nations S, Vadan R, Dai L, Luo M, et al Effects of functionalized and non- functionalized single-walled carbon nanotubes on root elongation of select crop species. Environ. Toxicol Chem. 2008;27:1922–31. 19086209 10.1897/08-117.1

[86] Chen R, Ratnikova TA, Stone MB, Lin S, Lard M, Huang G, et al Differential uptake of carbon nanoparticles by plant and mammalian cells. Small. 2010;6(5):612–7. 10.1002/smll.200901911 20209658

[87] Ghormade V, Deshpande MV, Paknikar KM. Perspectives for nano-biotechnology enabled protection and nutrition of plants. Biotechnology advances. 2011;29(6):792–803. 10.1016/j.biotechadv.2011.06.007 21729746

[88] Sayes CM, Fortner JD, Guo W, Lyon D, Boyd AM, Ausman KD, et al The differential cytotoxicity of water-soluble fullerenes. Nano Lett. 2004;4:1881–7.

[89] Nel A, Xia T, Madler L, Li N. Toxic potential of materials at the nanolevel. Science. 2006;311:622–7. 10.1126/science.1114397 16456071

[90] Trpković A, Todorović-Marković B, Trajković V. Toxicity of pristine versus functionalized fullerenes: mechanisms of cell damage and the role of oxidative stress. Arch. Toxicol. 2012;86:1809–27. 10.1007/s00204-012-0859-6 22562437

